# Front-of-pack nutrition label stimulates healthier product development: a quantitative analysis

**DOI:** 10.1186/1479-5868-7-65

**Published:** 2010-09-08

**Authors:** Ellis L Vyth, Ingrid HM Steenhuis, Annet JC Roodenburg, Johannes Brug, Jacob C Seidell

**Affiliations:** 1 Department of Health Sciences and the EMGO Institute for Health and Care Research, VU University Amsterdam, De Boelelaan 1085, 1081 HV Amsterdam, The Netherlands; 2 Department of Epidemiology and Biostatistics, EMGO Institute for Health and Care Research, VU University Medical Center, Van der Boechorststraat 7, 1081 BT Amsterdam, The Netherlands

## Abstract

**Background:**

In addition to helping consumers make healthier food choices, front-of-pack nutrition labels could encourage companies to reformulate existing products and develop new ones with a healthier product composition. This is the largest study to date to investigate the effect of a nutrition logo on the development of healthier products by food manufacturers.

**Methods:**

A total of 47 food manufacturers joining the Choices Foundation in the Netherlands (response: 39.5%) indicated whether their Choices products were newly developed, reformulated or already complied with the Choices criteria and provided nutrient composition data for their products (n = 821; 23.5% of the available Choices products in August 2009).

**Results:**

Most products carrying the logo as a result of reformulation and new product development were soups and snacks. Sodium reduction was the most common change found in processed meats, sandwiches, soups and sandwich fillings. Dietary fiber was significantly increased in most newly developed Choices product groups; for example, in fruit juices, processed meats, dairy products, sandwiches and soups. Saturated fatty acids (SAFA) and added sugar were significantly decreased both in reformulated and newly developed dairy products. Caloric content was significantly decreased only in reformulated dairy products, sandwich fillings and in some newly developed snacks.

**Conclusions:**

The results indicate that the Choices logo has motivated food manufacturers to reformulate existing products and develop new products with a healthier product composition, especially where sodium and dietary fiber are concerned.

## Background

The World Health Organization recommends limiting the intake of sodium, sugar, saturated fatty acids (SAFA) and trans fatty acids (TFA) in order to reduce the prevalence of diet-related chronic diseases [1]. The food industry, retailers and catering organizations can help consumers make healthy choices by offering products with reduced levels of these nutrients. Food reformulation and the development of new products with a favorable nutrient composition could assist with this.

A front-of-pack nutrition label can encourage food manufacturers to reformulate their products and develop new products with a favorable composition which would carry the label. Many countries have developed their own labels; for example, there is the Green Keyhole Symbol in Sweden [2], the Heart Symbol in Finland [3], the Multiple Traffic Light system and the Guideline Daily Amount in the United Kingdom [4], the Pick the Tick logo in Australia and New Zealand [5], and the Nuval system [6], the Guiding Stars symbol [7], and the Smart Choices program in the United States [8]. Although these nutrition labels have different designs and different product criteria, they generally have the same two aims: to help consumers make healthier food choices and to encourage food manufacturers to develop healthier products.

In the Netherlands the Choices nutrition logo has been found on a variety of products since 2006, available in many supermarket chains and food service locations including railway stations and worksite cafeterias. The criteria for the Choices logo were developed and are periodically adjusted by an independent scientific committee of experts in food and consumer behavior. The logo is assigned to products that contain lower levels of sodium, sugar, SAFA and TFA and caloric content and increased levels of dietary fiber compared with similar products within the same product category. A detailed background of the Choices logo has been described elsewhere [9,10].

Research indicates that the people who are health-conscious not only reported to purchase but also actually purchased more logo products [10,11]. The increased availability of healthier products, such as those carrying the logo, can be an efficient way to improve the diets of all consumer groups, whether or not they identify as health-conscious consumers. To date, only one study has evaluated the impact of a front-of-pack nutrition label on healthier product development [12]. This study, conducted in New Zealand, found that the Tick logo effectively influenced the food industry to reduce sodium levels in breakfast cereals, breads and margarines. In the Netherlands, it is assumed that the Choices logo has provided a clear incentive to companies, driving food reformulation and development in a healthier direction. Evidence for this, however, is lacking. Therefore, the aim of this study is to investigate the effect of the Choices logo on product reformulation and the development of new products with a favorable product composition. The following research questions were formulated:

- In which product groups are the most products reformulated or newly developed to comply with the Choices criteria?;

- Which nutrients have been changed in the reformulation process to comply with the Choices criteria and how much have these nutrients changed?; and

- What is the difference between the product compositions of newly developed Choices products and reference products that do not carry the logo?

## Methods

### Data collection

Between May 2007 and August 2009 all of the food manufacturers participating in the Choices program in the Netherlands (n = 119) were approached via email and phone and asked to participate in the study; 47 were willing to participate (response rate: 39.5%; main reason for non-response was lack of time). Participants were asked to complete an electronic questionnaire about their products carrying the Choices logo. First, they were asked to list the names of their Choices products and the corresponding product groups, as defined in the Choices program: vegetables and fruits (fresh, processed or juices), carbohydrates (processed or unprocessed potatoes, bread or grain products), proteins (meat, fish, eggs or meat substitutes (fresh or processed), dairy products, cheese products), oils and fats, ready-to-eat dishes, sandwiches, soups, sauces (water-based, emulsions or other sauces), snacks, beverages and other products (the background of the product groups has been explained elsewhere [12]). Furthermore, the food manufacturers were asked why each product had obtained the Choices logo. The following answer categories were provided:

a) Product already existed on the market and complied with the Choices criteria;

b) Existing non-complying product was reformulated to comply with the Choices criteria; or

c) A new product was developed that complied with the Choices criteria.

Additionally, for each Choices product manufacturers were asked to provide the product composition for energy density (kcal/100 g), SAFA (g/100 g), TFA (g/100 g), added sugar (g/100 g), sodium (mg/100 g), dietary fiber (g/100 g) and, if applicable, portion size (g). Food companies that had reformulated their products were asked to provide data on both pre-reformulation product composition and current (Choices-compliant) product composition. The companies returned product information on 878 products. Product information for 57 of these products was incomplete or the product was not available on the Dutch market, resulting in 821 useable products for further calculations and analyses. Because food manufacturers are allowed to assign the logo to fresh fruits and vegetables without changing their product composition, no data about fresh fruits and vegetables were collected. The study's protocol was approved by the Scientific Ethics Committee of VU University Amsterdam before the start of data collection and all food manufacturers provided written approval to use their data for scientific purposes.

### Statistical analysis

#### All products

Descriptive analysis was used to report the total number of products per product group which were reported to be newly developed, reformulated or already compliant with the Choices criteria.

#### Reformulated products

To estimate the effect of reformulation paired sample t-tests were used to explore differences in product composition per product group before and after reformulation. Product groups containing less than five reformulated products were considered to constitute too small a sample and consequently omitted from the analyses (e.g. beverages, ready-to-eat meals, water-based sauces, oils and fats).

#### Newly developed products

There is no pre-reformulation product composition reference for newly developed Choices products. Therefore, it was decided to use the same reference products for the analyses of the newly developed products that were used for the analyses of the reformulated products. For example, the mean product composition of the 68 pre-reformulated soups was used as the reference for the 21 newly developed Choices soups. In this case we assume that soups have a general pre-Choices product composition represented by the group of 68 soups. Independent sample t-tests were conducted to explore the differences in product composition between newly developed Choices products and reference products. Product groups containing less than five newly developed products and product groups lacking reference products were omitted from the analyses (e.g. potatoes, bread, cheese products, ready-to-eat meals, sauces, oils and fats).

Because most newly developed Choices products seemed to be snacks, extra analyses were conducted to determine their product composition. The snacks were divided into subgroups based on product type (fruit drink snacks, licorice, non-dairy ices, ice creams and savories) and their caloric content per portion was compared with the caloric content of reference products derived from the Dutch Food Composition Database [13], using one-sample t-tests. Subgroups containing less than five snacks were omitted from the analyses (peppermints). All statistical analyses were performed with the Statistical Package for the Social Sciences (SPSS version 17.0, 2009, Chicago, IL), and a significance level of 0.05 was adopted.

## Results

### All products

A total of 47 companies participated in the study, including one retailer and two caterers. Data were collected on 821 products, which was 23.5% of the total number of Choices products available on the market in August 2009 (excluding fresh fruits and vegetables). A total of 417 products were found to be existing products that complied with the Choices criteria; 168 products had been reformulated; 236 products were newly developed to comply with the Choices criteria. The number of Choices products produced by each company ranged from one to 300.

**Figure 1:**
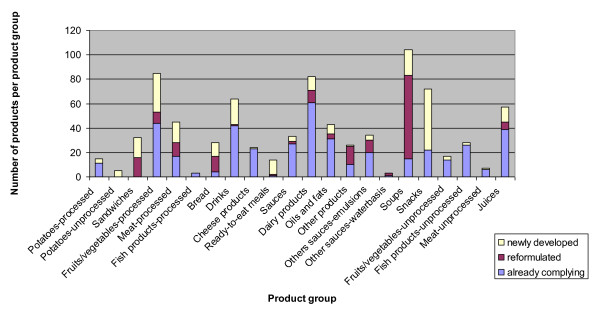
**Total number of products per product group that were newly developed, reformulated or already compliant with the Choices criteria**.

Figure 1 shows the total number of Choices products per product group, subdivided into existing compliant, reformulated and newly developed Choices products. Most products carrying the logo as a result of reformulation were soups (n = 68), followed by sandwiches (n = 16), other products (n = 15) and processed meat (n = 11). Most products carrying the logo as a result of new product development were snacks (n = 50), followed by processed fruits and vegetables (n = 32), fruit juices (n = 32), drinks (n = 21) and soups (n = 21).

### Reformulated products

Table 1 shows the product composition per product group before and after reformulation. Fiber levels in fruit juices were found to be significantly increased to obtain the Choices logo (*p *< 0.05). Sodium levels and SAFA were significantly reduced in the processed meats (*p *< 0.01 and *p *< 0.05, respectively). In dairy products, SAFA, added sugar and calories were found to be significantly reduced because of the logo criteria (all *p *< 0.05). Sodium levels in sandwiches were significantly reduced (*p *< 0.05), and fiber levels were increased (*p *< 0.01). Sodium was also significantly decreased in soups (*p *< 0.01). In sauces (emulsions), added sugar was decreased (*p *< 0.05). For sandwich fillings, SAFA (*p *< 0.01), TFA (*p *< 0.01), sodium *p *< 0.05) and calories (*p *< 0.01) were found to be decreased to obtain the logo.

**Table 1:** Mean (SD) nutrient content of reformulated products (Reform) and the pre-reformulation products (Previous) per product group^1^.

Product Category	**SAFA**^**a **^**Previous** (g/100g)	**SAFA**^**a **^**Reform** (g/100g)	**TFA**^**b **^**Previous** (g/100g)	**TFA**^**b **^**Reform** (g/100g)	Added Sugar Previous (g/100g)	Added Sugar Reform (g/100g)	Sodium Previous (mg/100g)	Sodium Reform (mg/100g)	Fiber Previous (g/100g)	Fiber Reform (g/100g)	Energy Previous (kcal/100g)	Energy Reform (kcal/100g)
Fruit juices (n = 6)	-	-	-	-	-	-	1.67 (0.52)	1.67 (0.52)	0.15 (0.12)	0.23* (0.18)	40.50 (3.62)	38.83 (5.49)

Processed meats (n = 11)	3.09 (2.46)	1.75* (0.71)	0.081 (0.163)	0.022 (0.031)	1.69 (0.95)	1.00 (0.82)	1017.82 (175.74)	834.55** (56.63)	0.07 (0.13)	0.14 (0.19)	242.82 (210.81)	237.73 (216.64)

Dairy products (n = 10)	1.26 (0.52)	0.88* (0.27)	-	-	5.74 (5.49)	1.46* (2.35)	50.30 (15.94)	52.80 (14.76)	-	0.18 (0.57)	57.10 (17.12)	51.20* (10.77)

Sandwiches (n = 16)	1.87 (1.76)	1.26 (0.86)	0.111 (0.207)	0.044 (0.053)	0.29 (0.68)	0.33 (0.82)	470.99 (295.55)	273.02* (96.34)	2.40 (1.04)	3.64** (0.86)	198.71 (61.44)	179.18 (28.76)

Soups (n = 68)	0.58 (0.48)	0.58 (0.48)	0.016 (0.019)	0.016 (0.019)	0.69 (0.71)	0.69 (0.71)	372.42 (47.91)	322.01** (32.11)	0.30 (0.44)	0.30 (0.44)	41.02 (18.46)	41.02 (18.46)

Sauces emulsions (n = 10)	2.54 (1.29)	2.77 (1.46)	0.253 (0.281)	0.168 (0.095)	6.12 (1.84)	5.31* (1.70)	0.71 (0.08)	0.69 (0.07)	1.51 (2.41)	1.51 (2.41)	220.00 (77.60)	215.00 (75.61)

Sandwich fillings (n = 8)	2.50 (0.53)	1.59** (0.73)	0.025 (0.004)	0.015** (0.005)	5.69 (2.01)	6.06 (2.21)	668.50 (311.29)	406.50* (117.84)	1.05 (0.46)	0.92 (0.62)	293.63 (53.36)	206.75** (49.86)

^1 ^Paired sample t-tests were used to explore differences in product composition per product group before and after reformulation.

^a ^SAFA: saturated fatty acids ^b ^TFA: trans fatty acids *p< 0.05 ** *p*< 0.01

### Newly developed products

Table 2 shows the product composition of the newly developed Choices products per product group (mean (SD)) compared with the reference products. The fiber levels in the fruit juices were found to be significantly higher compared to the reference products (*p *< 0.05). For processed meats, sodium levels were found to be significantly lower and fiber levels higher (all *p *< 0.01). In dairy products, SAFA (*p *< 0.01) and added sugar (*p *< 0.01) were significantly lower and fiber levels were higher (*p *< 0.05) than the reference product compositions. Fiber levels were also significantly higher in sandwiches, but added sugar levels were also found to be higher (all *p *< 0.05). For soups, sodium was significantly lower and fiber was higher (all *p *< 0.01). For all product groups, caloric values were found to be unchanged.

**Table 2:** Product composition of newly developed Choices products (New) per product group (mean (SD)) compared with reference products (Ref)^1^

Product Category	**SAFA**^**a **^ Ref (g/100g)	**SAFA**^**a **^ New (g/100g)	**TFA**^**b **^ Ref (g/100g)	**TFA**^**b **^ New (g/100g)	Added Sugar Ref (g/100g)	Added Sugar New (g/100g)	Sodium Ref (mg/100g)	Sodium New (mg/100g)	Fiber Ref (g/100g)	Fiber New (g/100g)	Energy Ref (kcal/ 100g)	Energy New (kcal/ 100g)
Fruit Juices (Ref: n = 6, New: n = 12)	-	-	-	-	-	2.13 (3.45)	1.67 (0.52)	2.58 (1.51)	0.15 (0.12)	0.40* (0.23)	40.50 (3.62)	43.00 (9.82)

Processed meat (Ref: n = 11, New: n = 17)	3.09 (2.46)	1.67 (0.73)	0.081 (0.163)	0.044 (0.043)	1.69 (0.95)	1.08 (1.10)	1017.82 (175.74)	626.04** (242.95)	0.07 (0.13)	0.41* (0.45)	242.82 (210.8)	191.02 (188.65)

Dairy products (Ref: n = 10, New: n = 11)	1.26 (0.52)	0.15** (0.18)	-	-	5.74 (5.49)	- **	50.30 (15.94)	46.09 (10.90)	-	0.52* (0.63)	57.10 (17.12)	63.45 (35.74)

Sandwiches (Ref: n = 16, New: n = 16)	1.87 (1.76)	2.08 (0.98)	0.111 (0.207)	0.095 (0.073)	0.29 (0.68)	1.80* (2.11)	470.99 (295.55)	358.97 (76.30)	2.40 (1.04)	3.19* (0.78)	198.71 (61.44)	209.25 (37.76)

Soups (Ref: n = 68, New: n = 21)	0.58 (0.48)	0.41 (0.34)	0.016 (0.019)	0.036 (0.026)	0.69 (0.71)	0.54 (0.52)	372.42 (47.91)	279.57** (63.40)	0.30 (0.44)	0.77** (0.60)	41.02 (18.46)	42.47 (21.34)

^1 ^The product composition of the reformulated products before reformulation was used as the reference. Independent sample t-tests were conducted to explore the differences in product composition between newly developed Choices products and reference products

^a ^SAFA: saturated fatty acids ^b ^TFA: trans fatty acids *p < 0.05 ** *p *< 0.01

**Figure 2:**
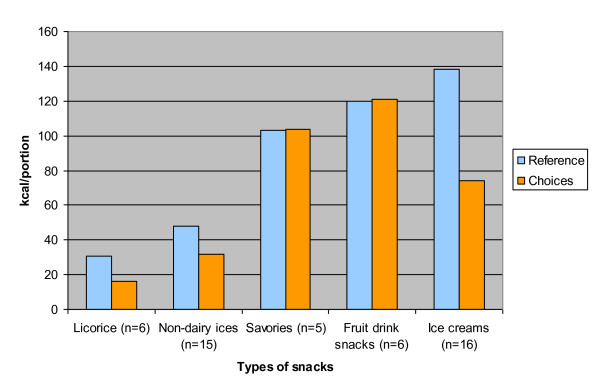
**Caloric content of the subgroups of newly developed Choices snacks compared with reference snacks**. The subgroups of Choices snacks and their selected reference products, derived from the Dutch Food Composition Database (2006) and portion sizes, were the following: - Choices: licorice - reference: licorice average (10 g). - Choices: non-dairy ices - reference: non-dairy ices (53 g). - Choices: savories - reference: prepared croquette (44 g). - Choices: fruit drink snacks - reference: fruit 2/day (200 ml). - Choices: ice creams - reference: vanilla ice cream (72 g). The caloric content of Choices snacks was compared with the reference products using one-sample t-tests. The caloric content of all subgroups of Choices snacks were found to be significantly lower than the reference snacks, with the exception of fruit drink snacks and savory snacks, which remained the same.

Figure 2 shows the caloric content per portion of newly developed Choices snacks compared with the reference snacks selected from the Dutch Food Composition Database [13]. The caloric content of all subgroups of Choices snacks was found to be significantly lower than the reference snacks, with the exception of fruit drink snacks and savory snacks, which remained the same.

## Discussion

To the best of our knowledge, this is the largest study to date to investigate the effect of a front-of-pack nutrition label on the development of healthier food products. Our data showed that most products carrying the logo as a result of reformulation and of new product development were soups and snacks, respectively. Sodium was the nutrient reformulated in the most products groups, namely in processed meats, sandwiches, soups and sandwich fillings. Dietary fiber was significantly higher in most newly developed Choices product groups when compared with reference products, namely in fruit juices, processed meats, dairy products, sandwiches and soups.

The finding that sodium is an important nutrient for reformulation is in agreement with a study from New Zealand that showed that the Pick the Tick logo effectively reduced the sodium content in a relatively small sample of food products [12]. In addition to a reduction in sodium, our study showed that the Choices logo also led to an improvement in the other nutrients with defined Choices criteria. For example, SAFA and added sugar were significantly decreased in both reformulated and newly developed dairy products. Dietary fiber was increased, also in product groups for which no fiber criteria were defined, such as processed meats and dairy products, possibly due to technological reasons. Newly developed Choices sandwiches, however, had a significantly higher sugar content than reference sandwiches, possibly to compensate for changes in other nutrients; this change deserves attention from a health perspective. Further, we only found significant reductions in the caloric content of dairy products and sandwich fillings after reformulation, and reductions in the caloric content of some newly developed snacks compared with reference snacks. The lack of major reductions in energy density is somewhat disappointing because a high intake of energy-dense food products is one of the major contributors to the prevalence of obesity [1]. Nevertheless, even small changes in calories can have a far-reaching public health impact. Roodenburg and colleagues showed a potential reduction in nutrient intakes, including calories, with the consumption of a diet complying with the Choices criteria, indicating their potential impact on energy balance [14]. This study is further discussed below.

Most newly developed Choices products were found in the category of snacks. Although the consumption of a limited number of snacks is promoted in the Netherlands, around 30% of a person's daily energy intake comes from food consumption between meals, and the greater part of that amount is snacks [15]. Our study showed that Choices snacks generally have a lower caloric content than regular snacks. Other nutrients were found to be changed in positive directions as well, such as decreased levels of SAFA in ice creams (milk-based) and decreased levels of sodium in licorice. This stresses the importance of further encouraging food manufacturers to develop healthier snacks. It has been debated whether it is justifiable to assign a health logo to snacks since the logo could stimulate snack consumption, which could constitute a negative side effect of the logo. Steenhuis and colleagues, however, showed that the use of the Choices logo had no negative side effects on the consumption of a chocolate mousse cake among females in a university community when they compared a cake with the logo to the same cake without it [16]. Nevertheless, it is of interest to note that the chocolate mousse cake was not perceived as healthy in that study. Other research indicates that the perception that a snack food was healthy did increase the actual intake of the food [17].

Our study does have some limitations. First, one could question whether our data can be considered a representative sample of the total number of Choices logo products available on the market. By collecting data from food manufacturers representing different types of industries, including multinationals, medium and small enterprises, retailers and caterers, we tried to create a sample that was as representative as possible and we did collect data from all product groups. Nevertheless, we did not collect enough data to be able to analyze all product groups, such as breads for example. Future research should try to include data on the product reformulation of breads because this category is the major source of sodium intake in the Netherlands and, therefore, is regarded as an important product for reformulation [18]. Secondly, it should be noted that some nutrients in quite a few product groups had a large standard deviation, due to the large variety of products within those product groups. Thirdly, the reference values for the newly developed Choices products could have been selected differently. It is possible that the food manufacturers developed new Choices products (for example, mango yogurt) based on existing non-Choices products (for example, strawberry yogurt) which were only slightly different from the Choices guidelines (for example, less sugar was added to the mango yogurt than to the strawberry yogurt, making the mango yogurt compliant with the Choices criteria). It could be useful for future food reformulation studies to ask food manufacturers more extensive questions about the composition development of newly introduced products. In this way, more valid reference values could be obtained (in this example the strawberry yogurt would have been the reference product for the newly developed Choices mango yogurt).

Finally, we collected data on a voluntary basis and all nutrient composition data were self-reported by the food manufacturers. The response rate was quite low and it is possible that only motivated food manufacturers participated in our research, especially those manufacturers that had significantly improved their products. Unfortunately, no data were collected about how many unhealthy products, or those not meeting the Choices criteria were introduced during the same time frame, to be able to evaluate the overall picture of the food supply. Nevertheless, the finding that motivated food manufacturers improved their products can be considered a positive starting point for the improvement of the availability of healthy products for consumers. It would be interesting for further research to explore why some food manufacturers are motivated to improve their products and others are not, and which aspects of company policies play a role in these decisions.

Despite these limitations, this is the largest study to date to explore the impact of a front-of-pack nutrition label on the development of healthier food products. Whether all significant changes can be considered nutritionally relevant remains to be determined. No consumption data and sales data were collected for this study. Consequently, we are only able to relate our findings to individual product groups and cannot make statements about the actual impact of the Choices logo on a population's health outcomes. Nevertheless, consuming a Choices-compliant diet could *potentially *lead to substantial improvements in nutrient intake, as reported by Roodenburg and colleagues [14]. In this study, the researchers combined food composition data and food consumption data and calculated the usual nutrient intake distributions in the Dutch population of young adults. Additionally, food products not complying with the Choices criteria were replaced by products that did comply. As a result, nutrient intakes for energy, total fat, SAFA, TFA, sodium, and total sugar decreased and fiber intake increased (these are the nutrients included in the Choices criteria). Additionally, positive changes were found for protein, total carbohydrate, PUFAs, MUFAs, calcium, iron and folic acid (nutrients not included in the Choices criteria). The challenge now is how to investigate the *actual *effect of the Choices logo by combining reformulation data with intake data and sales data. Consequently, possible health gains can be estimated, such as the prevalence of cardiovascular disease, life expectancy and health care costs. For example, in the United States, the Coronary Heart Disease Policy Model has been used to estimate the cost-effectiveness of a population-wide dietary salt reduction [19]. In future studies, using such a model could be helpful in estimating the impact of a front-of-pack nutrition logo on a population's health outcomes.

## Conclusions

This study indicates that the Choices logo has influenced food manufacturers to reformulate existing products and develop new products with a healthier product composition, especially where sodium and dietary fiber are concerned. Future studies should combine innovation data with consumption data and sales data to explore the impact of the Choices logo on a population's health outcomes.

## Competing interests

AJCR is seconded at VU University Amsterdam and employed by Unilever R&D, the Netherlands. The other authors have no conflicts of interest. This study was conducted by VU University Amsterdam and was funded by the Choices Foundation. The Choices Foundation played no role in the study design, analysis or interpretation of the data.

## Authors' contributions

ELV developed the design, collected and analyzed the data and wrote the manuscript. IHMS and AJCR developed the design, supervised data collection and data analyses and helped draft the manuscript. JB and JCS reviewed and critiqued the manuscript. All authors approved the final version of the manuscript.
